# Cannabidiol and nano-selenium mediate intestinal barrier function by affecting mucosal microstructures, and gut-associated immunological and oxidative stress response in the gut of chickens infected with *C. perfringens*

**DOI:** 10.3389/fimmu.2025.1529449

**Published:** 2025-04-28

**Authors:** Dominika Szkopek, Marta Mendel, Misza Kinsner, Katarzyna Ognik, Natalia Szyryńska, Bogdan Lewczuk, Krzysztof Kozłowski, Ivica Kos, Paweł Konieczka

**Affiliations:** ^1^ Department of Animal Nutrition, The Kielanowski Institute of Animal Physiology and Nutrition, Polish Academy of Sciences, Jabłonna, Poland; ^2^ Division of Pharmacology and Toxicology, Institute of Veterinary Medicine, Warsaw University of Life Sciences, Warsaw, Poland; ^3^ Department of Biochemistry and Toxicology, Faculty of Animal Sciences and Bio-Economy, University of Life Sciences in Lublin, Lublin, Poland; ^4^ Department of Histology and Embryology, Faculty of Veterinary Medicine, University of Warmia and Mazury, Olsztyn, Poland; ^5^ Department of Poultry Science and Apiculture, University of Warmia and Mazury in Olsztyn, Olsztyn, Poland; ^6^ Department of Animal Science and Technology, Faculty of Agriculture, University of Zagreb, Zagreb, Croatia

**Keywords:** cannabidiol, nano-selenium, *C. perfringens*, necrotic enteritis, gut barrier, DNA changes, broiler chickens

## Abstract

Nutritional additives with biological activity, such as cannabidiol (CBD) and nano-selenium (nano-Se), are viable to prevent bacterial diseases such as necrotic enteritis in chickens. The present study hypothesized that CBD and nano-Se mediate epigenetic and oxidative DNA changes in blood and intestinal epithelial cells and can affect intestinal development and functionality in broiler chickens at an early stage of infection with *C. perfringens*. This study revealed that both compounds, in combination under physiological or pathophysiological conditions, can act synergistically, improving the indices of histomorphometry of duodenum, jejunum, and ileum. Examination of the structures and ultrastructures of the gastrointestinal tract showed that CBD + nano-Se supplementation did not manifest adverse effects on the host gut indices. In contrast, epigenetic and oxidative markers of blood and gut structures indicated that these components balanced the immune system, mitigating the excessive inflammatory response caused by infection, which boosted the immune response of birds to challenge. There were also significant correlations between indicators of intestinal barrier function, such as diamine oxidase and lactic acid levels, and histomorphometry and markers of DNA integrity in the blood and intestine of chickens. In addition, it was shown that nano-Se increased hemoglobin concentration, which may be beneficial in the host's response to pathogen stimuli. These findings evidenced the health-promoting effect of cannabidiol and nano-selenium in *C. perfringens-*infected chickens and provided new insights into the mechanism of action of both nutritional additives.

## 1 Introduction

The gastrointestinal tract (GIT) plays a crucial role in the digestion and absorption of nutrients and defense against external agents. Intestinal integrity is key for maintaining the physical barrier between the intestinal lumen and the body and protecting against infection. The intestinal barrier comprises several parts, among which physical, chemical, and immunological components can be distinguished. The first layer is the extrinsic mucus layer, which consists of an outer layer associated with bacteria and an inner layer with high concentrations of secretory immunoglobulin A (IgA, SIgA) and mucin (1). The inner layer is adjacent to the second layer of the intestinal barrier, the intestinal epithelial cells (IEC), which is the single layer of epithelial cells, which are connected by tight junctions and separating the intestinal lumen from the underlying lamina propria (2, 3). IECs are the primary type of cells that come into contact with the external environment and are the primary component of innate immunity within the intestinal lymphoid tissue. They are, therefore, able to recognize pathogens through the release of antimicrobial molecules, the expression of innate immune receptors, and the secretion of a wide range of enzymes, neurotransmitters, hormones, as well as cytokines and chemokines that link innate and adaptive immune responses (1, 4). Enterocytes in the apical epithelium are responsible for nutrient absorption. Under normal physiological conditions, the integrity of the intestinal barrier remains intact, but direct or indirect damage to the IEC can cause disruption and leakage. Consequently, mucosal immune homeostasis is disrupted, nutrient absorption is impaired, and bacteria or their toxins, as well as other antigens, are translocated to the whole body of the host (5, 6).

In the early period of chicken growth in the farm conditions, disturbances in gastrointestinal function are common. GIT is considered the main entry point for pathogenic bacteria causing dysbiosis. Bacterial pathogens can impair intestinal barrier function by disrupting tight junctions (TJP) and initiating inflammatory cascades. Most attack epithelial cells via effector proteins or by producing endotoxins (7). Enteric diseases are a serious problem for the poultry industry due to reduced bird welfare, increased risk of contamination of animal products intended for human consumption, production losses, and increased mortality. Clinical enteropathies are estimated to constitute up to 50% of pathologies in broilers (8). Importantly, bacterial infections can contribute to epigenetic changes and modifications leading to DNA damage of different cells. They also exert immunosuppressive effects, induce reactions leading to oxidative stress in cells, and reduce the activity of antioxidant enzymes, which can result in the oxidation of lipids, proteins, and DNA and impair antioxidant defense mechanisms (9–11). Negative oxidative and epigenetic changes can induce intestinal inflammation, which, in turn, can directly disrupt the integrity of the intestinal barrier (12–14). Necrotic enteritis (NE), caused by the anaerobic bacterium *Clostridium perfringens* (*C. perfringens*), is a widespread disease among poultry worldwide. Over the past few years, the subclinical form of NE has become more common (15, 16). In this form, there are usually no visible clinical signs. Still, there is ongoing damage to the intestinal mucosa, leading to reduced digestion, absorption, and weight gain, as well as increased feed conversion ratio. Damage to the intestines can allow bacteria to penetrate the portal vessels and, consequently, the parenchymal organs in the body, disrupting many biochemical processes. NE induces oxidative processes associated with impaired intestinal absorption and compromised intestinal barrier integrity (7). Although the subclinical form does not cause a mortality rate as high as the acute form, it is just as dangerous because it can persist in flocks without manifesting symptoms (17). Consequently, it goes undiagnosed, resulting in some of the largest economic losses in the poultry industry (16, 18).

After the preventive use of antibiotics in livestock was prohibited in the European Union (Regulation 1831/2003/EC on additives for use in animal nutrition), nutritional supplements with biological activity have become viable to prevent bacterial diseases such as NE. Broilers are kept commercially for a short time, and as a result, potential interventions to help chickens recover from advanced NE during the final growth period are rare. Bioactive ingredients with potential modulatory activity determining the health status of GIT include cannabidiol (CBD) from hemp plants (*Cannabis sativa*), and nano-selenium (nano-Se) might be considered to prevent or ameliorate initial processes determining the pathogenicity of different agents. Interest in CBD is supported by evidence of its potential therapeutic value. Among its actions are anti-inflammatory, antioxidative, and immunomodulatory effects. CBD's actions are mediated by its influence on many molecular targets, including receptors, enzymes, transporters, or ion channels (19). Specifically, CBD exhibits a multitude of effects by influencing neurotransmitter release, cell signaling, gene expression and protein levels, cell cycle control, oxidative stress, and inflammation (20–22). The polypharmacology of CBD may explain its ability to be effective against diverse pathologies by recruiting different mechanisms depending on the system compromised in the given condition (20–22). Thus, epigenetic pathways induced by cannabinoids may play a key role in regulating inflammation by these compounds both endogenously and exogenously (23). Another compound showing biological activity is selenium. Selenium directly affects aspects such as oxidative stress, immune function, and survival (24–26). As an antioxidant, it protects cells by binding to free radicals and neutralizing active oxygen species (27). Additionally, it exhibits an indirect regulatory effect on GIT by modulating the protective host response. Selenium supplementation also enhances the production of key components of innate immunity (28, 29). Importantly, selenium nanoparticles (nano-Se) have higher bioavailability but lower toxicity than selenium in other forms (30, 31). Both CBD and nano-Se show positive effects on chickens challenged with *C. perfringens* (28, 32, 33). The beneficial effects of both agents are manifested in increased expression of genes determining intestinal barrier function and levels of antibodies against toxins, altered intestinal bacterial enzyme activity, and increased collagenase activity. Importantly, CBD and nano-Se in chickens with NE show a mitigating effect due to protection against DNA damage to the intestinal mucosa through changes in the activity of DNA damage repair enzymes (34). To the best of the authors’ knowledge, no assessment of epigenetic and oxidative DNA changes in blood and intestinal epithelial cells or assessment of gastrointestinal structures has been performed to date in chickens at the early stage of *C. perfringens* infection when supplemented with CBD and nano-Se. The current study also verified changes at the ultrastructural level in the gut to investigate the molecular actions of the host’s response to challenges and the activity of bioactive compounds in mediating this response. Previous reports indicate that changes at the ultrastructural level may play a key role in initiating the gut-associated immune systems due to stress stimuli in chickens (35–37). Given the above, it is hypothesized that cannabidiol and nano-selenium mediate epigenetic DNA changes, immunological and oxidative stress response in blood and GIT, and can affect intestinal barrier development and functionality in early post-infection broiler chickens.

## 2 Materials and methods

### 2.1 Animal experiment

#### 2.1.1 Bioethics

The Local Ethical Committee for Animal Research at UWM Olsztyn has approved this study (Resolution No. 54/2019 of 30 July 2019). All procedures involving animals were conducted in accordance with ARRIVE guidelines, EU Regulations (Directive 2010/63/EU), Polish Law, and the Declaration of Helsinki.

#### 2.1.2 Birds, housing, experimental design and diets

**Figure 1:**
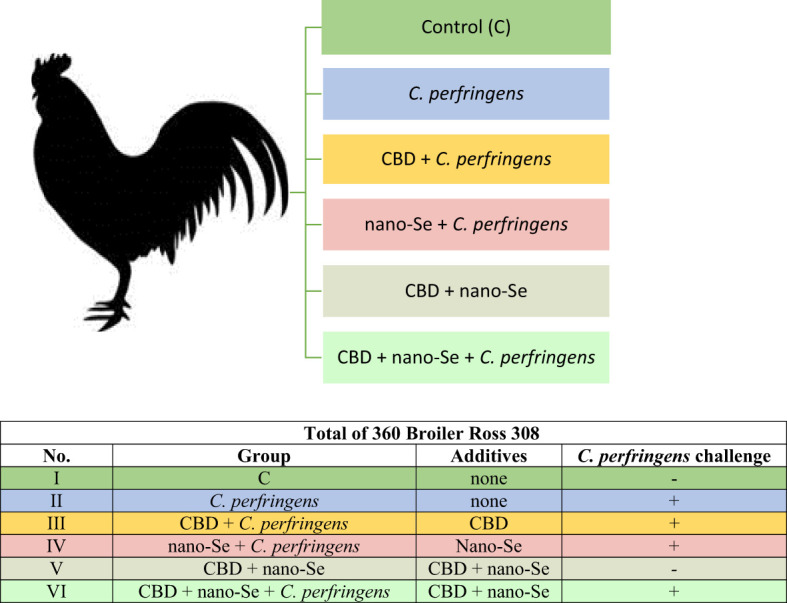
The division into experimental groups.

**Figure 2:**
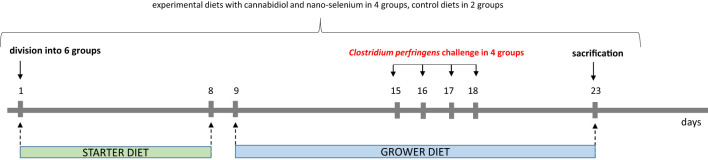
Study design.

A total of 360 one-day-old male broiler chicks were purchased from a local hatchery. Upon arrival, the broilers were divided into six treatment groups with eight replicate cages of nine chickens per group according to average body weight. The conditions, such as temperature, light cycle and humidity, were maintained according to standard management practices for commercial chicken houses. Birds on days 0–8 were fed a starter diet, and a grower diet on days 9-23, which was formulated to meet or exceed the requirements for broiler chickens. The basal grower diet was based on wheat (50.76%), soybean meal (21.76%), triticale (15.54%), fish meal (5.18%), and rapeseed meal (4.15%) (32), which made the gut environment favorable for *C. perfringens* growth. Birds in the negative control (I, C) and positive control (II, *C. perfringens*) groups consumed a basal diet throughout the experiment. Groups CBD + *C. perfringens* (III) and nano-Se + *C. perfringens* (IV) were challenged and supplemented (on top) with 15 g/kg *Cannabis sativa* extract or with 0.3 mg/kg nano-Se. Chicks from CBD + nano-Se (V) group and CBD + nano-Se + *C. perfringens* (VI) were fed a control grower diet supplemented with both additives, but the second group was challenged with *C. perfringens*. The division into groups is shown in **Figure 1**. A simple study design is shown in **Figure 2**.

#### 2.1.3 Chemical composition of cannabidiol and nano-selenium

Nano-selenium was used in the form of a nanopowder with an average particle size of 10–45 nm, specific surface area (SSA) of approximately 30–50 m^2^/g, and purity of 99.9%, according to the manufacturer’s declaration (American Elements, CA, USA) (32). Hemp panicles (*Cannabis sativa*) were obtained from plants at the Institute of Natural Fibers and Medical Plants in Poznan, Poland. The supercritical carbon dioxide extract of hemp was obtained from the Institute of New Chemical Synthesis, Pulawy, Poland. The extraction parameters were followed according to the previous protocol (32). Hemp extract contained 12% CBD, 0.49% tetrahydrocannabinol and 0.38% tetrahydrocannabinolic acid (32).

#### 2.1.4 *Clostridium perfringens* challenge and sample collection

Before the *C. perfringens* challenge, 1 mL of a coccidial cocktail was administered to all birds at 14 and 15 days of age to create a favorable gut environment for *C. perfringens* colonization (32). The birds were given *C. perfringens* in four challenged groups at 15, 16, 17, and 18 days of age. Briefly, after 4 hours of food deprivation, animals in *C. perfringens*, CBD + *C. perfringens*, nano-Se + *C. perfringens*, and CBD + nano-Se + *C. perfringens* groups were given (*per os*) 1 mL of inoculum (brain–heart infusion medium) containing approximately 10^8^ CFU/mL *C. perfringens* type A strain 56 bacteria, which was isolated from infected chickens, according to the previous protocol (32). The birds in the C and CBD + nano-Se groups were each administered 1 mL of sterilized broth medium.

On day 23, the birds were weighed, and eight broilers from each group were electrically stunned (150 mA, 350 Hz) and decapitated. Blood samples were collected into test tubes with an anticoagulant (heparin) from the wing vein in two replicates. One sample was for the determination of hemoglobin in whole blood, and the other sample was centrifuged at 3000 × g for 10 min, and blood plasma was collected. Subsequently, the entire digestive tract was removed from the same birds, and 1 cm long sections of the duodenum (on the flexion), jejunum (at Meckel's diverticulum) and ileum (5 cm proximal of the ileocecal junction) were collected and placed in buffered 10% formalin solution and in a mixture of paraformaldehyde and glutaraldehyde in phosphate buffer for histological analyses. In addition, the samples of jejunum and blood were immediately frozen at -80^o^C for ELISA analysis.

### 2.2 Histometric analysis of the duodenum, jejunum and ileum

Segments of the duodenum, jejunum, and ileum were fixed immediately after collection according to the laboratory procedure (38). After fixation, the material was dehydrated in EtOH solutions with increasing concentrations of 70%, 96%, and 99.8%. The material sections were then placed in xylene, impregnated with paraffin, and embedded in paraffin blocks (Para Plast Regular, P-3558, Merck KGaA, Darmstadt, Germany). The blocks were cut into 4.5 μm thick sections using an electric rotary microtome (Microm 350, Germany). The sections were placed on degreased and silane-coated (Menzel - Glaser, Germany) slides and allowed to dry for 24 hours at 42°C. Standard eosin/hematoxylin staining was used for histometric analysis of intestinal tissues. Images were analyzed using a light microscope (Olympus BX51 microscope; Olympus Corp., Tokyo, Japan) and CellD Imaging Software (Olympus Soft Imaging Solutions, Münster, Germany). The following parameters were measured: 1) villi height (VH), measured from the top of the villi to the villi-crypt junction, 2) crypt depth (CD), measured from the mouth of the crypts to their base, 3) villi width (VW), measured at the midline of the villi, 4) wall thickness (WT), measured from the top of the villi to the bottom of the muscular lamina of the mucosa, 5) villi area (VA) calculated as villus perimeter × VH, and 6) villi height to crypt depth ratio (VC). In addition to histometry, an evaluation of histological structures in the intestines was also performed. The images of the slides were prepared using a Pannoramic 250 Flash scanner (3DHistech, Budapest, Hungary).

### 2.3 Evaluation of ultrastructures in the jejunum

Gut samples were immediately fixed in a mixture of 1% paraformaldehyde and 2.5% glutaraldehyde in 0.2 M phosphate buffer for 2 hours at 4°C, then washed and post-fixed in 2% osmium tetroxide for 2 hours. After dehydration using ethanol (30, 50, 70, 80, 90, 95, 100%), the samples were embedded in Epon 812. Ultrathin sections were cut using the PT3D PowerTome ultramicrotome with ASH2 (Boeckeler Instruments, USA), placed on silicon wafers, and contrasted with uranyl acetate and lead citrate. Sections were imaged using a SenseBSD backscatter electrons detector in SEM Gemini 450 at 1.4 kV, controlled by Atlas 5 software (Carl Zeiss, Germany).

### 2.4 Immunoenzymatic analyses in blood and jejunum

The levels of 8-hydroxy-2-deoxyguanosine (8-OHdG), caspase 3 (Casp 3), diamine oxidase (DAO), lactic acid (LA), immunoglobulin A (IgA) and immunoglobulin Y (IgY) were determined in the wall of the jejunum and the blood using chicken ELISA kits (MyBioSource, San Diego, USA; Cat. No: MBS2464632, MBS261903, MBS044169, MBS2801903, MBS705241, MBS701964) with a microplate-reading spectrophotometer (Multiskan Sky, Thermo Scientific, Rockford, IL, USA). DNA was isolated from the blood and intestinal wall using QIAGEN kits (QIAGEN, Hilden, Germany). Epigenetic changes in the blood and intestinal wall were determined by analyzing global DNA methylation (methylome, 5-methylcytosine level) using Sigma Aldrich diagnostic kits (Sigma Aldrich, St. Louis, MO, USA; Cat. No: MDQ1-96RXN). The activity of superoxide dismutase (SOD) and glutathione peroxidase (GPx) in the blood and jejunum of broilers were determined by spectrometry using Ransel and Ransod diagnostic kits manufactured by Randox (Poland; Cat. No: SD125, RS505). Tumor necrosis factor α (TNF-α) and total antioxidant capacity (T-AOC) assessment in blood were carried out using chicken ELISA kits (MyBioSource, San Diego, USA; Cat. No: MBS746318, MBS2611919).

### 2.5 Blood hemoglobin content

Hemoglobin content (Hb) in the blood was determined using an Abacus Junior Vet hematology analyzer (Diatron, Budapest, Hungary).

### 2.6 Statistical analysis

Data are presented as the means (n=8 chickens per group), and variability is expressed as the pooled standard error of the mean (SEM) test or standard deviation (SD) values. Differences between groups were assessed using one-way ANOVA with the least significant difference (LSD) test. The significance level was set at P<0.05. Correlations between markers of jejunum function (DAO, LA), histomorphometry, and epigenetic and oxidative indicators (Casp3, GPx, IgA, IgY, SOD, 8-OHdH, % of methylated DNA) were assessed with Pearson correlation analysis. Statistical calculations were performed in STATGRAPHICS Centurion XVI ver. 16.1.03 software.

## 3 Results

### 3.1 Effect of treatment and challenge on gut histomorphometry

Parameters such as VH (µm), CD (µm), VW (µm), WT (µm), VA (mm^2^), and VC were measured in the chickens’ duodenum, jejunum, and ileum. The results of the histological measurements are presented in **Table 1**.

**Table 1:** Light microscopy evaluation of histomorphometry of the duodenum, jejunum and ileum.

Indices	I	II	III	IV	V	VI	SD	SEM	P-value
Duodenum									
Villi height, µm	1908.7	1949.3	1856.1	1707.3	1853.8	1898.0	83.824	29.974	0.262
Crypt depth, µm	171.5^a^	199.9^b^	192.8^b^	199.0^b^	161.3^a^	171.8^a^	16.527	3.039	0.001^*^
Villi width, µm	131.2^ab^	133.4^ab^	141.3^abc^	148.8^bc^	152.2^c^	124.9^a^	10.637	2.616	0.011^*^
Wall thickness, µm	170.9	137.5	141.8	139.4	171.1	138.6	16.418	4.749	0.067
Villi area, mm^2^	803.7	829.8	839.5	810.6	914.4	757.6	51.815	20.809	0.409
Villi height to crypt depth ratio	11.40^cd^	9.79^abc^	9.63^ab^	8.59^a^	11.56^d^	11.14^bcd^	1.193	0.271	0.003^*^
Jejunum									
Villi height, µm	1152.6^ab^	1151.8^ab^	1284.8^bc^	1031.0^a^	1379.8^c^	1331.1^bc^	131.888	29.079	0.001^*^
Crypt depth, µm	149.1^a^	194.2^b^	197.1^b^	188.5^b^	152.4^a^	151.2^a^	23.393	3.939	0.001^*^
Villi width, µm	149.0	140.1	136.3	139.3	134.2	128.9	6.741	1.920	0.058
Wall thickness, µm	139.6	149.6	135.3	131.0	142.2	118.5	10.659	3.230	0.098
Villi area, mm^2^	551.6	522.0	565.6	466.3	593.2	554.8	43.737	13.157	0.092
Villi height to crypt depth ratio	7.77^bc^	5.94^a^	6.52^ab^	5.50^a^	9.49^d^	8.93^cd^	1.634	0.303	0.001^*^
Ileum									
Villi height, µm	690.5	852.1	857.4	742.3	688.8	800.5	76.116	22.457	0.078
Crypt depth, µm	165.9	160.0	152.6	156.9	141.3	153.9	8.271	2.486	0.091
Villi width, µm	141.3^c^	131.6^bc^	129.0^bc^	126.0^b^	127.0^b^	109.5^a^	10.353	2.245	0.001^*^
Wall thickness, µm	156.6^c^	129.3^a^	150.2^bc^	134.7^ab^	131.2^ab^	138.8^abc^	10.963	2.850	0.021^*^
Villi area, mm^2^	324.9	369.7	368.7	305.6	287.7	284.0	38.319	12.285	0.144
Villi height to crypt depth ratio	4.17^a^	5.34^b^	5.58^b^	4.83^ab^	4.95^ab^	5.27^b^	0.498	0.158	0.135

The different letters indicate significant differences (P<0.05). ^*^ Significant differences. Variability is expressed as the pooled standard error of the mean (SEM) test and standard deviation (SD) values. Group designations: I control (C); II *C. perfringens*; III CBD + *C. perfringens*; IV nano-Se + *C. perfringens*; V CBD + nano-Se; VI CBD + nano-Se + *C. perfringens*

#### 3.1.1 Duodenum

Crypt depth was significantly higher in the *C. perfringens*, CBD + *C. perfringens*, and nano-Se + *C. perfringens* groups compared to the C, CBD + nano-Se and CBD + nano-Se + *C. perfringens* groups (P=0.001). There were no significant differences in crypt depth between the negative control, CBD + nano-Se, CBD + nano-Se + *C. perfringens*, and between positive control and infected groups with single additions of CBD or nano-Se. Villi width was significantly increased in the control group with CBD and nano-Se compared to the C, *C. perfringens*, and infected group with two additives and significantly decreased in the CBD + nano-Se + *C. perfringens* group compared to the nano-Se + *C. perfringens* and CBD + nano-Se groups (P=0.011). There was no significant difference between the control group and the infected group with CBD and nano-Se. Villi height to crypt depth ratio was significantly lower in the infected group with nano-Se compared to the control group and non-challenged and challenged groups with both additives and significantly higher in the CBD + nano-Se group compared to the infected control group and infected groups with CBD or nano-Se (P=0.003). No differences were found between the control group and the infected and challenged groups with both additives. There were no statistically significant differences in villi height, wall thickness, or villi area between the experimental groups (P>0.05).

#### 3.1.2 Jejunum

Villi height was significantly lower in the nano-Se + *C. perfringens* group compared to the CBD-challenged group and the challenged and not-challenged groups (CBD and nano-Se) and significantly higher in the group not-challenged with both additives compared to the positive and negative control groups and the nano-Se-challenged group (P=0.001). There was no significant difference between challenged and not-challenged groups with both additives. Crypt depths were significantly lower in the C, CBD + nano-Se, and CBD + nano-Se + *C. perfringens* groups compared to the *C. perfringens* group, and the infected groups challenged with CBD or nano-Se (P=0.001). There were no differences between the C, CBD + nano-Se, and CBD + nano-Se + *C. perfringens* groups or between the *C. perfringens* and infected groups supplemented with CBD or nano-Se. Villi height to crypt depth was significantly higher in the uninfected group challenged with both additives, and all groups except the infected group were fed diets with CBD and nano-Se additives (P=0.001). This ratio was significantly higher in the CBD + nano-Se + *C. perfringens* group compared to the infected group with or without CBD or nano-Se additives (P=0.001). There was no difference in the value of this ratio between the infected group without additives, and the groups with CBD or nano-Se. There were no statistically significant differences in villi width, wall thickness, and villi area between chickens from all various groups (P>0.05).

#### 3.1.3 Ileum

Villi width was significantly lower in the CBD + nano-Se + *C. perfringens* group compared to all other groups tested (P=0.001). This parameter in groups CBD + nano-Se and CBD + nano-Se + *C. perfringens* was significantly lower than in the control group (P=0.001). There were no significant differences between the control group and the groups challenged with CBD or nano-Se. Wall thickness was significantly lower in the infected control group than in the positive control group and the infected group with CBD addition, and these parameter values were significantly lower in groups IV and V than in group I (P=0.021). There were no significant differences in wall thickness values between the infected group and the nano-Se + *C. perfringens*, CBD + nano-Se, and CBD + nano-Se + *C. perfringens* groups. Villi height to crypt depth ratio was significantly higher in the *C. perfringens*, CBD + *C. perfringens*, and CBD + nano-Se + *C. perfringens* groups compared to the control group. There were no statistically significant differences in villi height, crypt depth, or villi area among the treated and non-treated groups (P>0.05).

### 3.2 Light microscopy evaluation of histology of the small intestine

**Figure 3:**
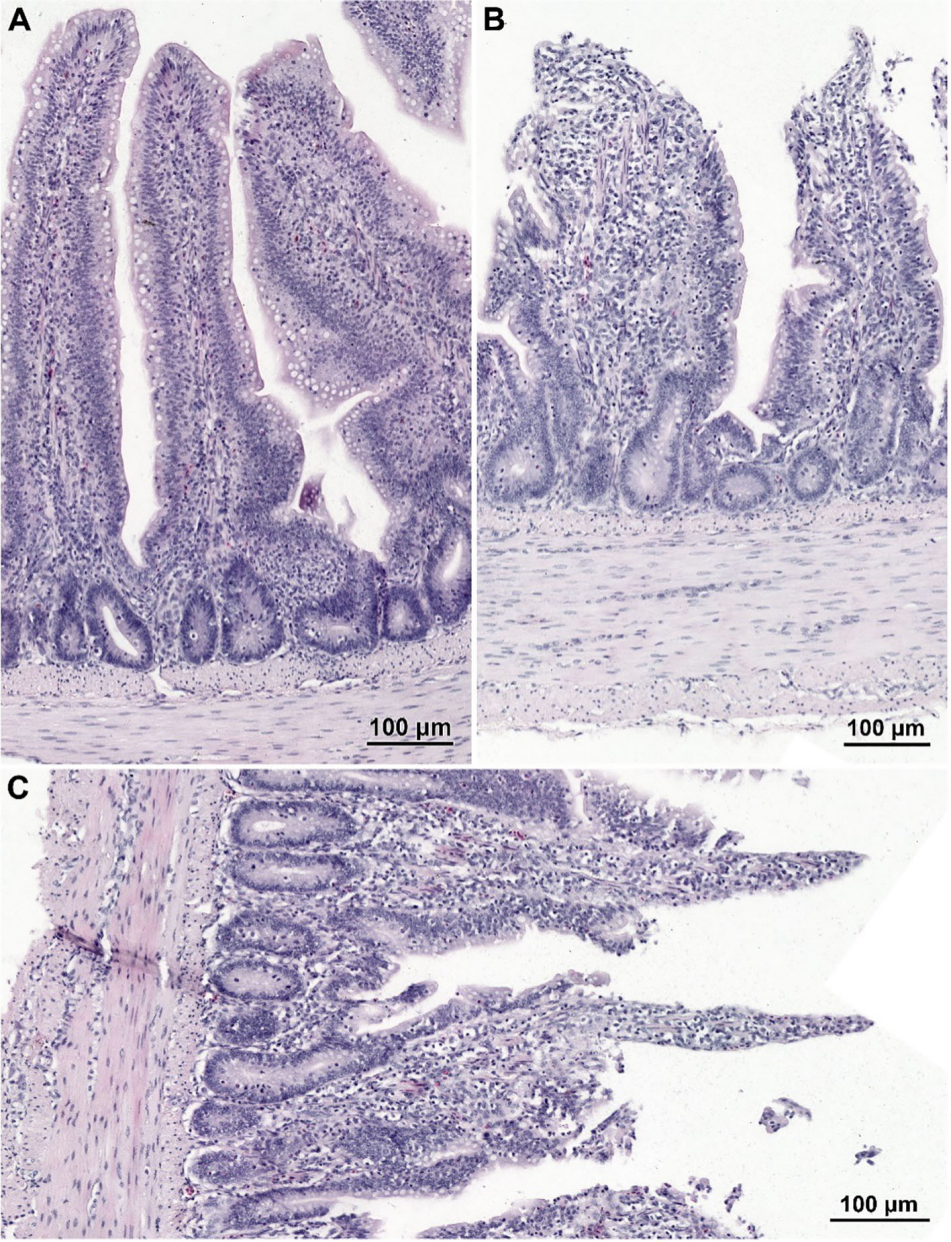
Histological structure of the jejunum in non-infected chicken receiving CBD + nano-Se **(A)** and birds infected with *C*. *perfringens* but not supplemented (with CBD and nano-Se) **(B, C)**. **(A)** Note well-developed villi covered by a continuous layer of columnar epithelial cells. **(B, C)** Note the lack of epithelium cover in the apical parts of villi and shortage of villi **(B)** and abnormal shape of villi **(C)**.

**Figure 4:**
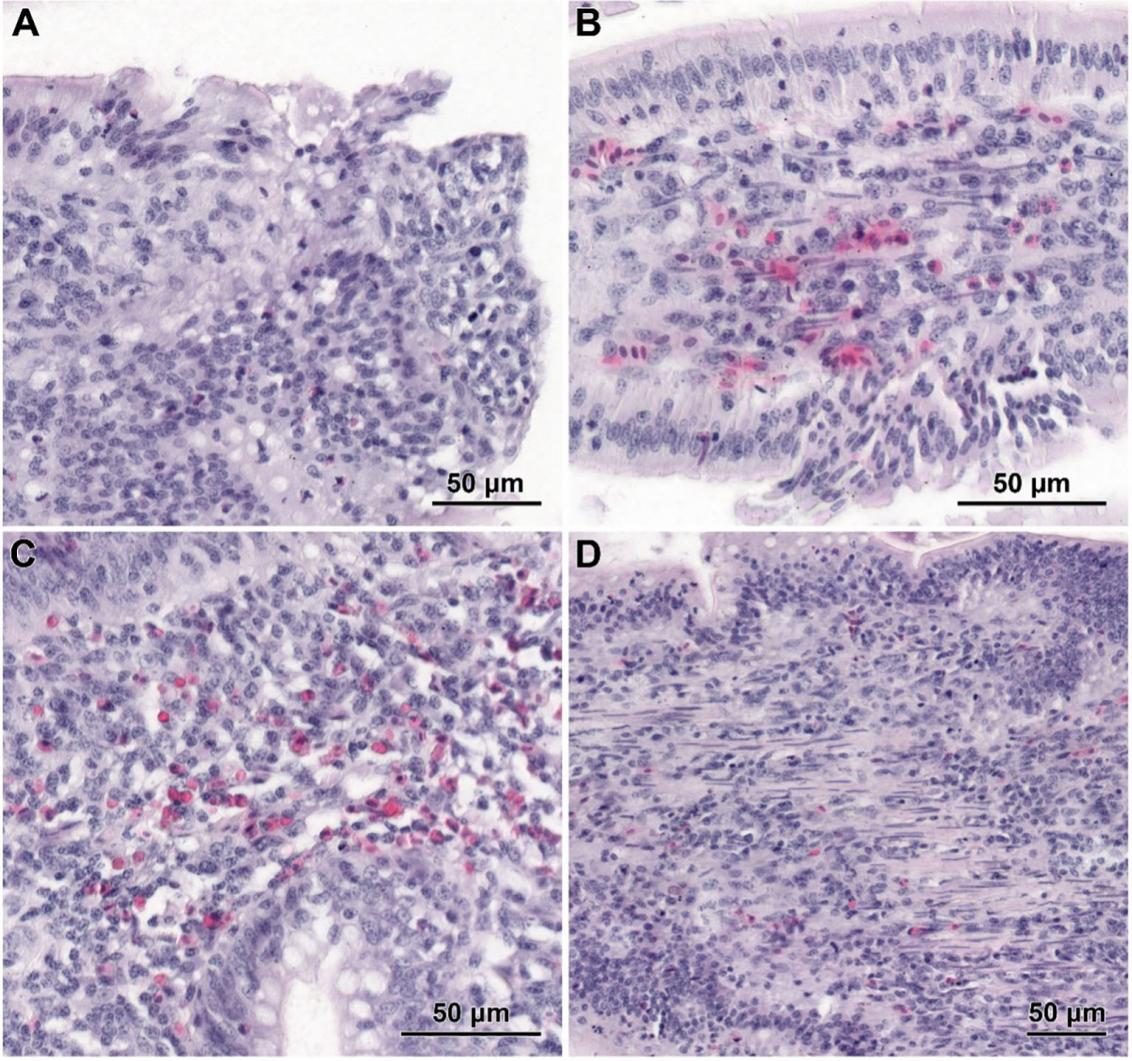
Histological structure of jejunum in *C*. *perfringens* infected chickens receiving CBD **(A)**, CBD + nano-Se **(B)**, and nano-Se **(C, D)**. **(A)** The lack of epithelial cells on the apical part of the villus. **(B)** Shedding of enterocytes from the latter part of the villus. **(C)** Infiltration of inflammatory cells inside the villus. **(D)** Increased presence of smooth muscle cells within the stroma of villi.

The histological structure of the small intestine in control chickens was typical of the species. Well-developed villi were observed, with the longest found in the duodenum and the shortest in the ileum, accompanied by short intestinal glands. The pathological alternations were not detected. No qualitative differences in villi structure were noted between infected groups treated with CBD, CBD + nano-Se, nano-Se, and those not supplemented with these compounds. The structure of the small intestine in animals receiving CBD + nano-Se was similar to that in the control group (**Figure 3A**). The villi were covered by a continuous layer of columnar cells comprising absorptive enterocytes and goblet cells. In contrast, all groups of birds infected with *C. perfringens* frequently exhibited areas of mucosa devoid of epithelial covering, primarily on the apical portions of villi (**Figures 3B, C**, **4A**). However, the shedding of enterocytes from lateral portions of the villi was also noted (**Figure 4B**). Occasionally, the loss of epithelial cells coincided with shortened villi (**Figure 3B**) or villi deformation (**Figure 3C**). Some villi displayed connective tissue stroma infiltrated by inflammatory cells (**Figure 4C**) and, in certain cases, an increased presence of smooth muscle cells (**Figure 4D**). Although the intestinal glands were not affected by the *C. perfringens* infection, mitotic figures appeared to be more numerous in infected chickens than in non-infected birds, except for the infected group treated with CBD + nano-Se.

### 3.3 The electron microscopy study of the jejunum

**Figure 5:**
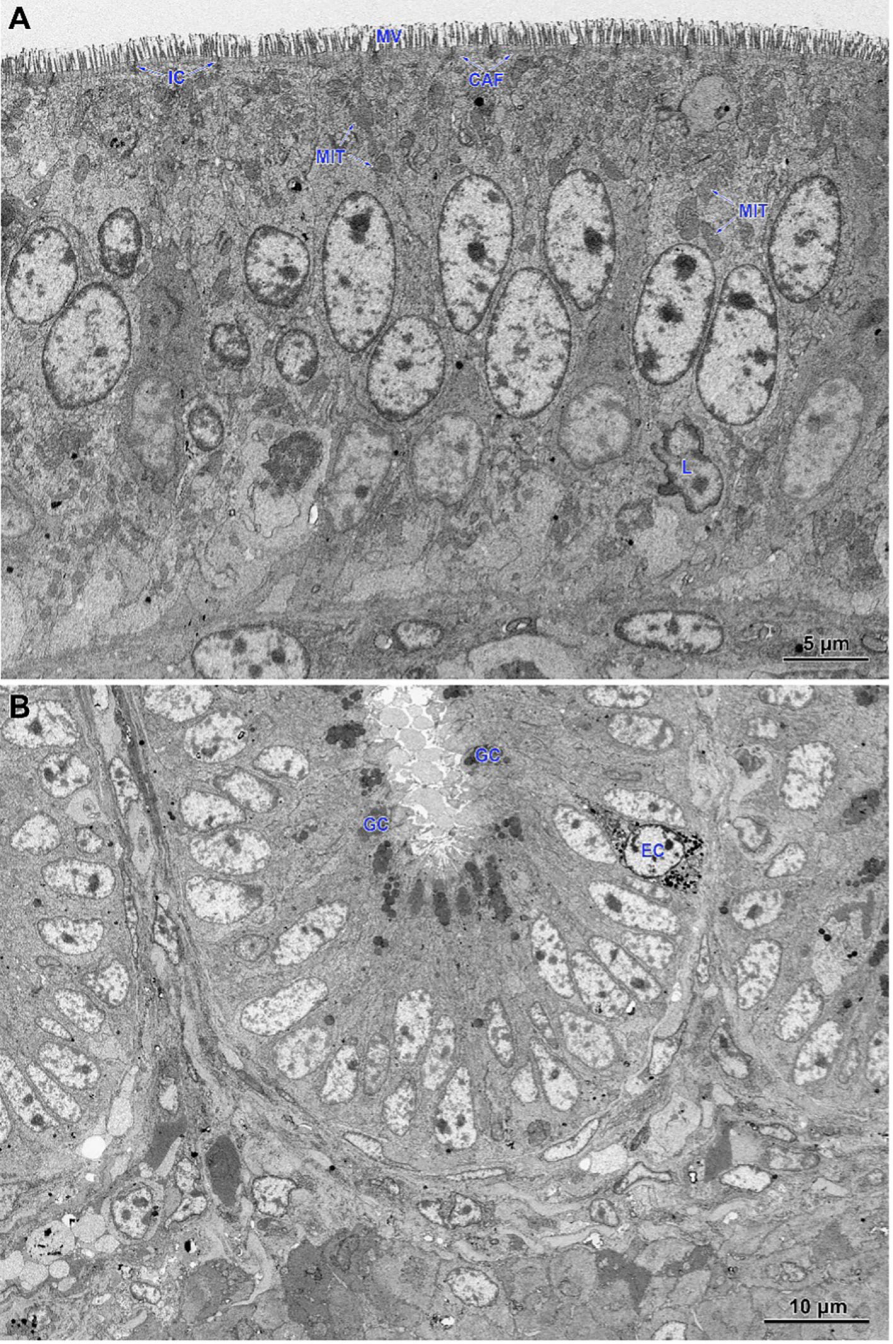
Ultrastructure of the jejunum of non-infected chicken receiving CBD + nano-Se. **(A)** Epithelium covering the villus. Note the regular shape and arrangement of cells. **(B)** The lower part of the intestinal gland is composed of immature cells. MIT, mitochondria; MV, microvilli; CAF, cortical actin filaments; IC, intercellular junctions; L, lymphocytes; GC, goblet cells; EC, enteroendocrine cells.

In non-infected chickens (groups C and CBD + nano-Se), the epithelium of the villi consisted of a continuous and regular layer of columnar cells connected via tight and anchoring junctions located near their apical poles (**Figure 5A**). Microvilli covering enterocytes were regularly distributed but not very densely packed. The layer of cortical actin filaments was well-developed. The apical parts of enterocytes comprise mitochondria with a matrix of moderate electron density, Golgi apparatus and both forms of the endoplasmic reticulum. Lipid droplets were sparse in these cells. The goblet cells contained large secretory granules. Lymphocytes were sparse to moderate in number. The intestinal glands comprise immature enterocytes and goblet cells, as well as enteroendocrine cells (**Figure 5B**). Injured cells were extremely rarely found.

**Figure 6:**
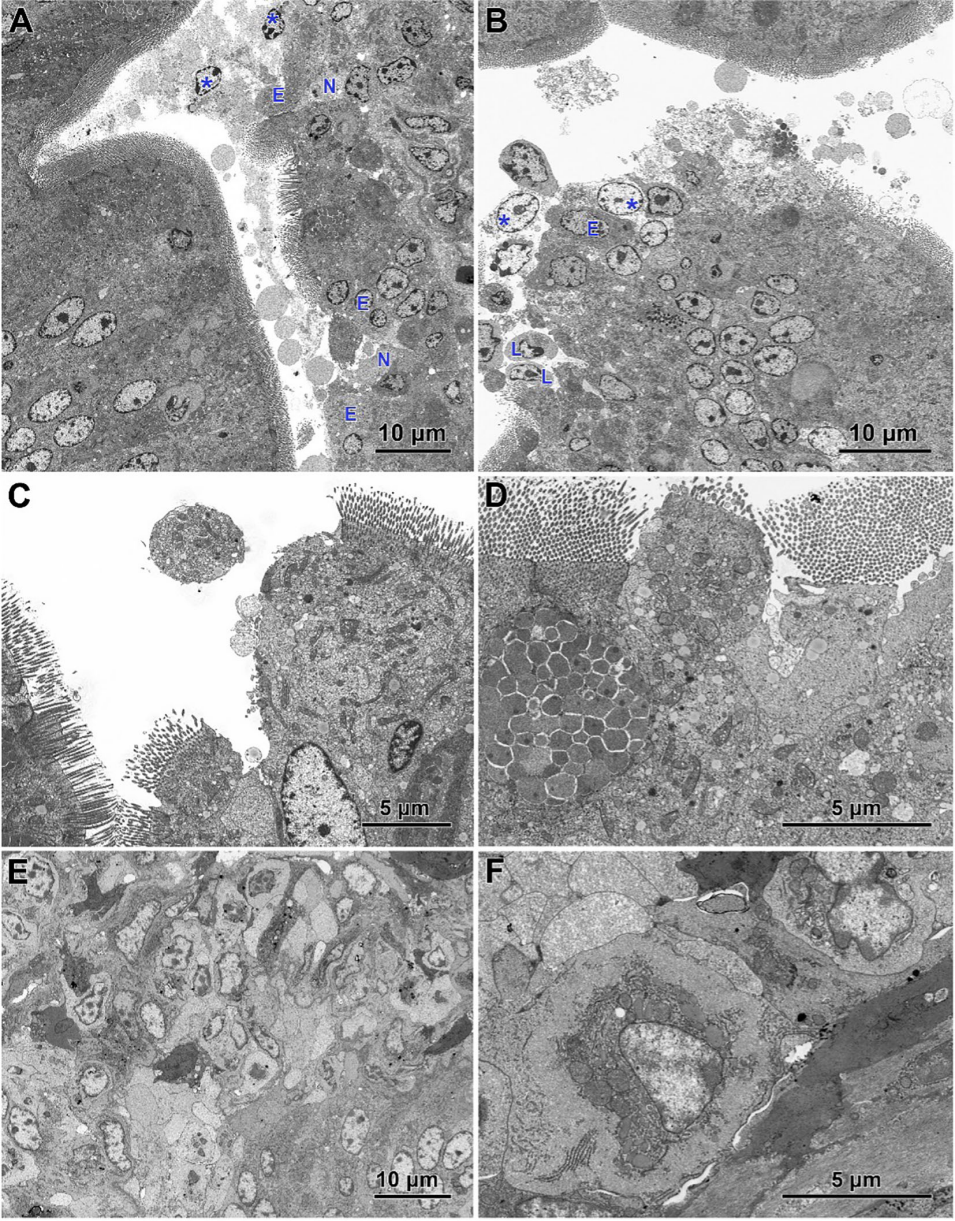
Ultrastructure of intestinal epithelium in the jejunum of chickens infected with *C*. *perfringens*. **(A)** Necrotic areas (N) containing necrotic cells and cellular debris (asterisks) as well as abnormal enterocytes (E) lacking the cell polarity and microvilli. **(B)** The apical parts of the villi with the injured epithelium cover. Note necrotic cells (asterisks), lymphocytes (L), and irregularly shaped enterocytes without microvilli (E). **(C, D)** Areas of abnormal epithelium. Note the irregular arrangement of enterocytes. **(E)** Inflammatory cells inside the villus. **(F)** Smooth muscle cells inside the villus.

Electron microscopy revealed the presence of small necrotic regions within the epithelium covering both the apical and lateral surfaces of the intestinal villi in chickens infected with *C. perfringens* (**Figure 6A**). In addition to necrotic cells and cellular debris, these regions comprised abnormal enterocytes lacking cell polarity and microvilli. The cell debris was also present in the intestine lumen over the necrotic regions. The apical portions of villi, described at light microscopy level as devoid of the epithelium cover, contained necrotic cells, cellular debris, and numerous inflammatory cells, but also irregularly shaped enterocytes without microvilli (**Figure 6B**). Enterocytes in these areas were loosely attached and did not form the tight junctions. Ultrastructural analysis also revealed areas where enterocytes formed a continuous or nearly continuous layer but were highly irregular in shape and devoid of microvilli (**Figures 6C, D**). The stroma of some villi contained numerous inflammatory cells, frequently with foamy cytoplasm (**Figure 6E**) and an increased presence of smooth muscle cells (**Figure 6F**).

**Figure 7:**
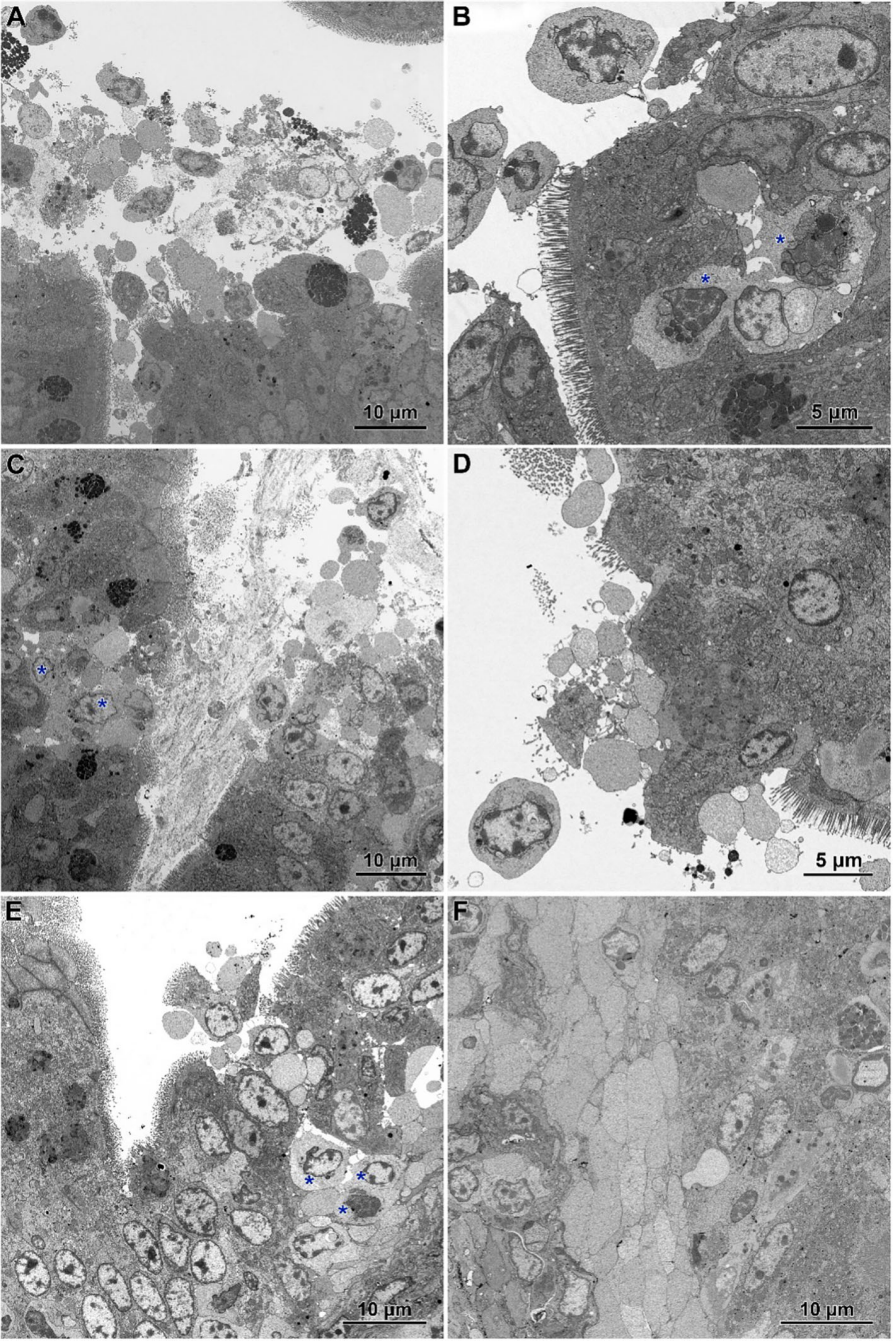
Ultrastructure of jejunum in *C. perfringens* infected chickens receiving CBD **(A, B)**, CBD + nano-Se **(C, D)**, and nano-Se **(E, F)**. **(A)** Necrotic cells and cellular debris over the injured part of the intestinal epithelium. **(B)** Abnormal epithelium with irregular-shaped enterocytes without microvilli and numerous inflammatory cells (asterisks). **(C)** Necrotic foci covered by cellular debris. Note inflammatory cells (asterisks). **(D)** Enterocytes with irregular shape and arrangement. Note the sparse presence of microvilli. **(E)** Infiltration of the injured epithelium by inflammatory cells (asterisks). **(F)** Inflammatory cells with foam cytoplasm inside the villus.

Infected chickens treated with CBD, CBD + nano-Se, or nano-Se displayed similar ultrastructural changes as those infected with *C. perfringens* but not supplemented with these compounds (**Figures 7A–F**). Necrotic foci covered with cellular debris (**Figures 7A, C**), areas of abnormal epithelium containing irregularly shaped enterocytes without microvilli (**Figures 7B–D**), intense infiltration of the epithelium by lymphocytes (**Figures 7B, E**), and the presence of inflammatory cells with foamy cytoplasm in the villi stroma (**Figure 7F**) were common across all infected groups receiving CBD, nano-Se, or CBD + nano-Se.

### 3.4 Epigenetic and oxidative changes as a response to treatment

#### 3.4.1 Blood

**Figure 8:**
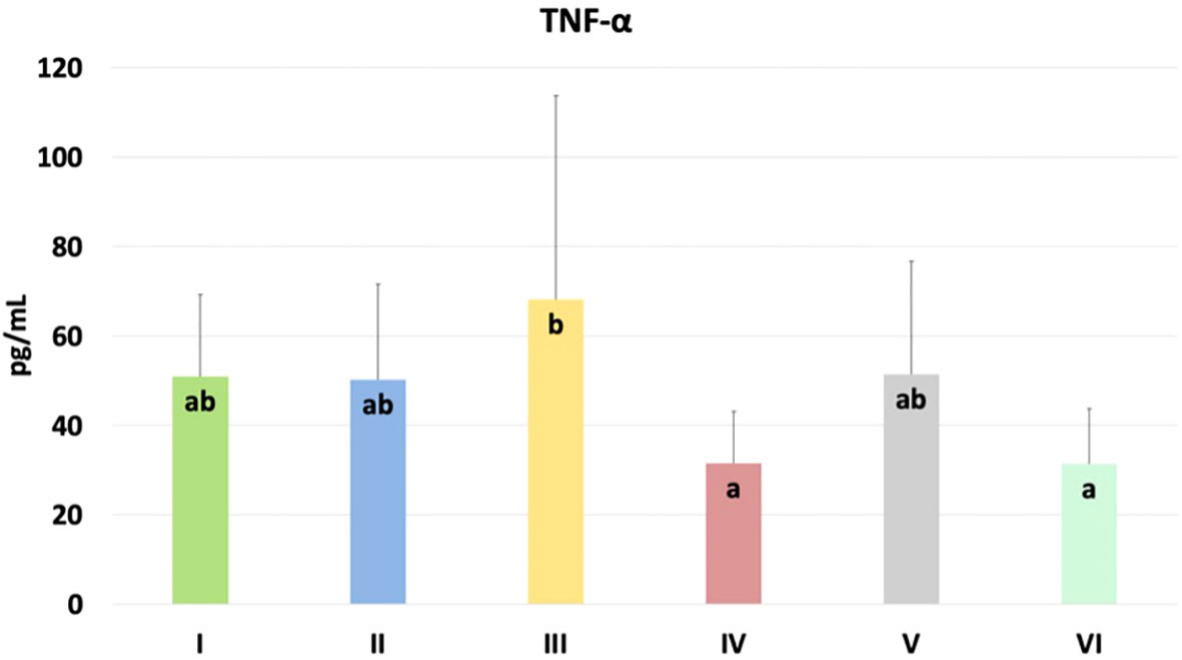
The TNF-α levels in the blood plasma of chickens. The different letters indicate significant differences (P<0.05). The error bars indicate the mean standard deviations (SD) for the eight chickens in each treatment group. Group designations: I control (C); II *C. perfringens*; III CBD + *C. perfringens*; IV nano-Se + *C. perfringens*; V CBD + nano-Se; VI CBD + nano-Se + *C. perfringens*.

The results of selected indicators of epigenetic and oxidative changes in chicken blood plasma are shown in **Table 2**. Significant differences in tumor necrosis factor-alpha (TNF-α) levels are shown in **Figure 8**. TNF-α levels were significantly higher in the group challenged with CBD, compared to the nano-Se + *C. perfringens* and CBD + nano-Se + *C. perfringens* groups (P=0.048). The difference between the group challenged with both additives and the C group was not significant. No significant differences were shown in the other assays.

**Table 2:** Epigenetic and oxidative changes in chicken blood.

Indices	I	II	III	IV	V	VI	SD	SEM	P-value
T-AOC [mmol/L]	2.147	2.090	2.077	2.054	2.084	2.040	0.037	0.018	0.643
Casp 3 [ng/mL]	0.095	0.132	0.126	0.098	0.085	0.137	0.022	0.008	0.250
GPx [ng/mL]	18.380	18.310	21.890	19.760	20.680	18.350	1.493	1.037	0.898
IgA [ng/mL]	0.121	0.042	0.043	0.059	0.069	0.059	0.029	0.009	0.102
IgY [ng/mL]	3.953	2.238	3.390	2.960	3.461	2.485	0.645	0.223	0.223
SOD [U/L]	248.800	334.600	323.600	338.500	298.400	309.700	33.093	15.150	0.572
TNF-α [pg/mL]	50.890^ab^	50.160^ab^	68.170^b^	31.550^a^	51.370^ab^	31.450^a^	13.946	3.898	0.048^*^
8-OHdG [ng/mL]	2.152	2.744	2.350	2.917	2.066	2.705	0.348	0.128	0.318
% of methylated DNA	41.950	46.930	47.670	49.150	48.160	69.490	9.610	2.872	0.087

The different letters indicate significant differences (P<0.05). ^*^ Significant differences. Variability is expressed as the pooled standard error of the mean (SEM) test and standard deviation (SD) values. Group designations: I control (C); II C + *C. perfringens*; III C + CBD + *C. perfringens*; IV C + nano-Se + *C. perfringens*; V C + CBD + nano-Se; VI C + CBD + nano-Se + *C. perfringens.* T-AOC, total antioxidant capacity; Casp 3, caspase 3; GPx, glutathione peroxidase; IgA, immunoglobulin A; IgY, immunoglobulin Y; SOD, superoxide dismutase; TNF-α, tumor necrosis factor-alpha; 8-OHdG, 8-Hydroxy-2'-deoxyguanosine.

#### 3.4.2 Jejunum

**Figure 9:**
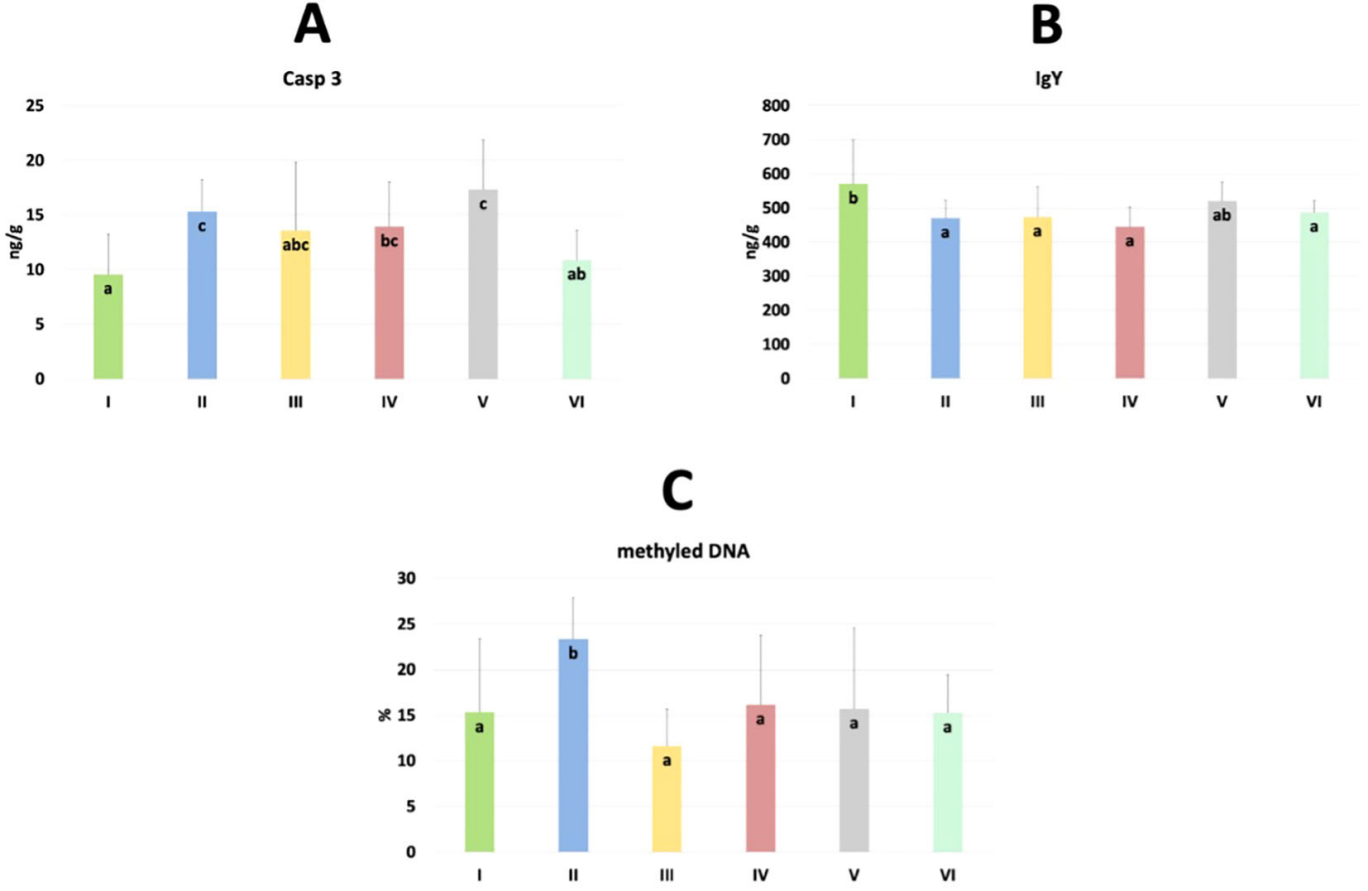
The levels of caspase 3 (Casp 3) **(A)**, immunoglobulin Y (IgY) **(B)** and % of methylated DNA **(C)** in the jejunum of chickens. The different letters indicate significant differences (P<0.05). The error bars indicate the mean standard deviations (SD) for the eight chickens in each treatment group. Group designations: I control (C); II *C*. *perfringens*; III CBD + *C*. *perfringens*; IV nano-Se + *C*. *perfringens*; V CBD + nano-Se; VI CBD + nano-Se + *C*. *perfringens.*.

The results of selected indicators of epigenetic and oxidative changes in chicken jejunum are shown in **Table 3**. Significant differences in caspase 3 (Casp 3), immunoglobulin Y (IgY), and % of methylated DNA levels are shown in **Figure 9**. Casp 3 levels were significantly higher in the *C. perfringens* and CBD + nano-Se groups compared to the C and CBD + nano-Se + *C. perfringens* groups and significantly lower in the control group compared to the *C. perfringens*, nano-Se + *C. perfringens* and CBD + nano-Se groups (P=0.008). IgY levels were significantly lower in the *C. perfringens*, CBD + *C. perfringens*, nano-Se + *C. perfringens*, and infected groups fed both additives compared to the C group (P=0.031). The % of methylated DNA was significantly higher in the control infected group compared to all other groups tested (P=0.029). No significant difference was found between group C and the group challenged with CBD and nano-Se in % of methylated DNA. No significant differences were found between the other markers.

**Table 3:** Epigenetic and oxidative changes in chicken jejunum.

Indices	I	II	III	IV	V	VI	SD	SEM	P-value
Casp 3 [ng/g]	9.576^a^	15.305^c^	13.569^abc^	13.920^bc^	17.322^c^	10.881^ab^	2.838	0.687	0.008^*^
GPx [ng/g]	46.700	52.560	41.210	45.180	42.030	40.040	4.626	1.061	0.474
IgA [ng/g]	2.081	1.625	2.096	1.874	2.030	1.643	0.214	0.120	0.768
IgY [ng/g]	570.900^b^	470.400^a^	473.600^a^	445.600^a^	520.900^ab^	487.300^a^	44.708	11.999	0.031^*^
SOD [U/g]	0.577	0.508	0.591	0.604	0.658	0.645	0.054	0.019	0.260
8-OHdG [ng/g]	25.193	22.284	24.272	25.401	23.288	22.759	1.292	0.902	0.900
% of methylated DNA	15.296^a^	23.349^b^	11.623^a^	16.149^a^	15.704^a^	15.283^a^	3.846	1.026	0.029^*^

The different letters indicate significant differences (P<0.05). ^*^ Significant differences. Variability is expressed as the pooled standard error of the mean (SEM) test and standard deviation (SD) values. Group designations: I control (C); II *C. perfringens*; III CBD + *C. perfringens*; IV nano-Se + *C. perfringens*; V CBD + nano-Se; VI CBD + nano-Se + *C. perfringens.* Casp 3, caspase 3; GPx, glutathione peroxidase; IgA, immunoglobulin A; IgY, immunoglobulin Y; SOD, superoxide dismutase; 8-OHdG, 8-Hydroxy-2'-deoxyguanosine.

### 3.5 Correlation between markers of intestine function in blood or gut tissue and histomorphometry

The correlation between diamine oxidase (DAO; U/L) and lactic acid (LA; mmol/L) in blood or intestine and histological measurements is shown in **Table 4**. In blood, a statistically significant negative correlation was found between the villi width of the duodenum and DAO (P=0.036), between LA and crypt depth of the duodenum (P=0.017), villi width of the jejunum (P=0.002), and villi width of the ileum (P=0.016). In the intestine, a statistically significant positive correlation was found between DAO and the villi height to crypt depth ratio (P=0.032), and between LA, wall thickness (P=0.007) and villi area (P=0.035) of the duodenum. In the jejunum, no significant correlation existed between measurements and the concentration of DAO and LA. However, in the ileum, there was a significant positive correlation between villi width (P=0.029) and crypt depth (P=0.029) and DAO and between villi width and LA (P=0.034). There was also a significant negative correlation between DAO concentration and villi height to crypt ratio in the ileum.

**Table 4:** Correlation between blood or intestine concentration of DAO and LA and gut histomorphometry.

Blood	DAO		LA	
Duodenum	*r*	P-value	*r*	P-value
Villi height, µm	0.093	0.528	-0.019	0.899
Crypt depth, µm	-0.060	0.677	-0.342	0.017^*^
Villi width, µm	-0.307	0.036^*^	-0.187	0.202
Wall thickness, µm	-0.077	0.604	-0.035	0.813
Villi area, mm^2^	-0.178	0.226	-0.167	0.258
Villi height to crypt depth ratio	0.114	0.441	0.242	0.097
Jejunum				
Villi height, µm	0.134	0.365	0.135	0.361
Crypt depth, µm	0.006	0.966	-0.095	0.519
Villi width, µm	-0.213	0.145	-0.442	0.002^*^
Wall thickness, µm	0.032	0.828	0.032	0.828
Villi area, mm^2^	0.024	0.873	-0.126	0.392
Villi height to crypt depth ratio	0.119	0.420	0.153	0.300
Ileum				
Villi height, µm	-0.059	0.691	0.030	0.841
Crypt depth, µm	0.006	0.970	-0.042	0.775
Villi width, µm	-0.177	0.230	-0.347	0.016^*^
Wall thickness, µm	0.007	0.965	-0.002	0.987
Villi area, mm^2^	-0.153	0.298	-0.167	0.258
Villi height to crypt depth ratio	-0.075	0.610	0.057	0.699

Intestine	DAO		LA	
Duodenum	*r*	p-value	*r*	p-value
Villi height, µm	0.101	0.496	0.157	0.288
Crypt depth, µm	-0.248	0.089	0.055	0.712
Villi width, µm	-0.007	0.962	0.266	0.068
Wall thickness, µm	-0.033	0.823	0.385	0.007^*^
Villi area, mm^2^	0.028	0.849	0.306	0.035^*^
Villi height to crypt depth ratio	0.310	0.032^*^	0.049	0.741
Jejunum				
Villi height, µm	-0.015	0.920	-0.206	0.160
Crypt depth, µm	-0.028	0.852	0.110	0.457
Villi width, µm	-0.041	0.783	0.137	0.352
Wall thickness, µm	-0.054	0.714	-0.046	0.758
Villi area, mm^2^	-0.041	0.784	-0.113	0.445
Villi height to crypt depth ratio	0.016	0.915	-0.236	0.106
Ileum					
Villi height, µm	-0.126	0.392	-0.0002	0.991
Crypt depth, µm	0.315	0.029^*^	0.113	0.443
Villi width, µm	0.315	0.029^*^	0.308	0.034^*^
Wall thickness, µm	0.181	0.219	0.197	0.179
Villi area, mm^2^	0.058	0.697	0.163	0.269
Villi height to crypt depth ratio	-0.297	0.041^*^	-0.062	0.675

Calculated Pearson’s correlation coefficient between markers of jejunum function levels in blood and histomorphometry of duodenum, jejunum and ileum. ^*^ Significant correlation at P<0.05. DAO, diamine oxidase; LA, lactic acid.

### 3.6 Correlation between markers of jejunum function and epigenetic and oxidative indicators

The correlation between diamine oxidase (DAO; U/L) and lactic acid (LA; mmol/L) in the intestine and levels of selected epigenetic and oxidative indicators in blood or jejunum are shown in **Table 5**. In blood, no statistically significant differences were found between indicator concentrations and intestinal DAO concentrations. In contrast, a statistically significant positive correlation was found between LA and SOD concentrations (P=0.015). In the gut, a statistically significant negative correlation was found between DAO and IgA (P=0.045) and a significant positive correlation between DAO and IgY (P=0.200). A significant positive correlation occurred between LA and 8-OHdG concentrations (P=0.036).

**Table 5:** Correlation between jejunum concentration of DAO and LA and epigenetic and oxidative indicators in blood or gut tissue.

Jejunum	DAO		LA	
Blood	*r*	P-value	*r*	P-value
Casp 3 [ng/g]	-0.211	0.151	-0.147	0.319
GPx [ng/g]	-0.123	0.406	0.107	0.468
IgA [ng/g]	-0.045	0.759	-0.022	0.880
IgY [ng/g]	0.002	0.987	0.016	0.914
SOD [U/g]	-0.058	0.696	0.351	0.015^*^
8-OHdG [ng/g]	0.034	0.821	0.035	0.811
% of methylated DNA	0.070	0.636	0.100	0.501

Jejunum	DAO		LA	
Jejunum	*r*	P-value	*r*	P-value
Casp 3 [ng/g]	0.050	0.737	0.135	0.360
GPx [ng/g]	0.123	0.406	0.184	0.212
IgA [ng/g]	-0.290	0.045^*^	-0.050	0.737
IgY [ng/g]	0.334	0.020^*^	0.161	0.276
SOD [U/g]	-0.020	0.894	-0.214	0.145
8-OHdG [ng/g]	0.019	0.897	0.303	0.036^*^
% of methylated DNA	0.074	0.616	0.017	0.907

Calculated Pearson’s correlation coefficient between epigenetic and oxidative indicators and markers of jejunum function levels in blood of chickens subjected to different treatments. ^*^ Significant correlation at P<0.05. DAO, diamine oxidase; LA, lactic acid; Casp 3, caspase 3; GPx, glutathione peroxidase; IgA, immunoglobulin A; IgY, immunoglobulin Y; SOD, superoxide dismutase; 8-OHdG, 8-Hydroxy-2'-deoxyguanosine.

### 3.7 Cannabidiol and nano-selenium affect hemoglobin content in chicken blood

**Figure 10:**
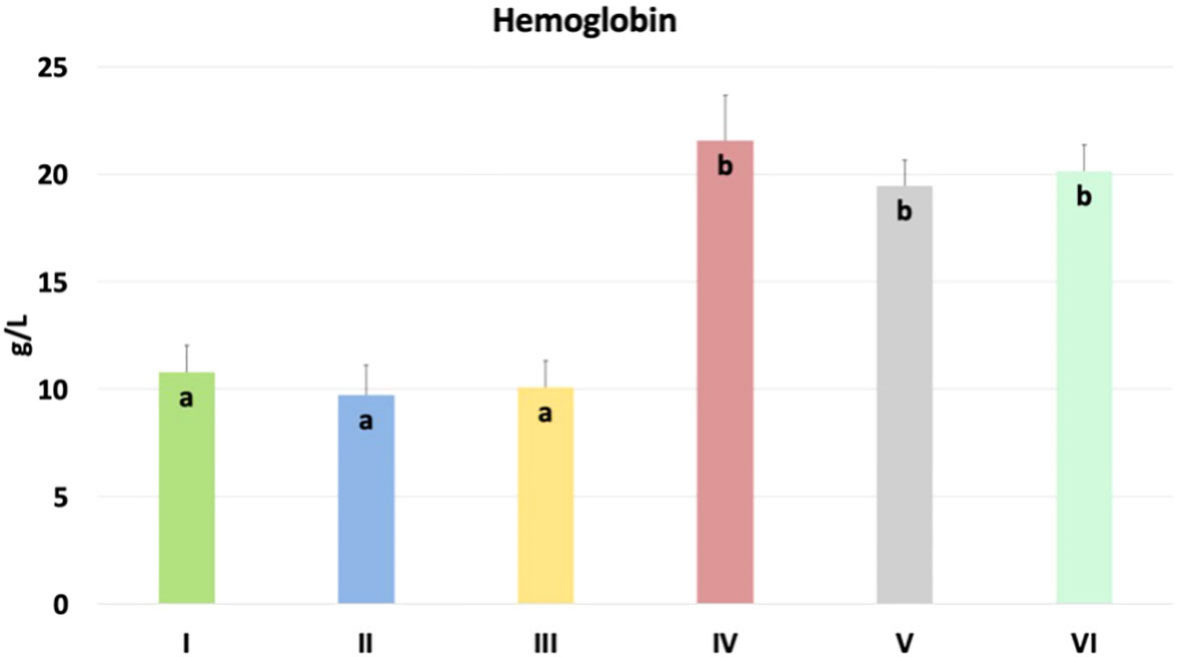
Hemoglobin content in chicken blood. The different letters indicate significant differences (P<0.05). The error bars indicate the mean standard deviations (SD) for the eight chickens in each treatment group. Group designations: I control (C); II *C. perfringens*; III CBD + *C. perfringens*; IV nano-Se + *C. perfringens*; V CBD + nano-Se; VI CBD + nano-Se + *C. perfringens*.

The results of the hemoglobin content in chicken blood are shown in **Figure 10**. Significantly higher content was found in nano-Se + *C. perfringens*, CBD + nano-Se, and CBD + nano-Se + *C. perfringens* compared to groups C, *C. perfringens*, CBD + *C. perfringens* groups (P=0.001). No significant differences were detected between negative and positive control groups and among CBD + *C. perfringens* groups, as well as between groups challenged with the addition of nano-selenium and infected and non-infected with both groups supplemented with additives.

## 4 Discussion

To the best of the authors’ knowledge, this is the first study to assess epigenetic and oxidative DNA changes occurring in the gut and blood and to examine in detail the structures and ultrastructures of the gastrointestinal tract in chickens challenged with *C. perfringens* bacteria and supplemented with cannabidiol and nano-selenium.

This study performed a histomorphometric analysis of the duodenum, jejunum, and ileum to assess the impact of infection and nutritional additives. Intestinal morphology is crucial for digestion and absorption, and indices such as villi height (VH), crypt depth (CD), villi width (VW), wall thickness (WT), villi area (VA), and villi height to crypt depth ratio (VC) are commonly used to assess intestinal health (39). However, limited evidence is available on how CBD affects gut function and anatomy in broiler chickens. This study shows that VH in the jejunum was significantly higher in the non-infected group with CBD and nano-Se supplementation compared to the control, *C. perfringens*, and infected group with nano-Se supplementation, and VH was lower in the nano-Se + *C. perfringens* group compared to the infected and non-infected group fed a diet with the two supplements. In contrast, neither treatment nor infection affected VH in the duodenum and ileum. As reported, longer villi are usually associated with improved absorption in the gut (40). In the case of VH, this result is consistent with the studies of Bami et al. (41) and Safdari et al. (42), who also showed that nano-selenium significantly increased VH. It can be concluded from this that cannabidiol may also have a gut-promoting effect. In contrast, the reduction in VH in the nano-Se + *C. perfringens* group compared to the infected group fed a diet with the two additives shows that, in this case, NE infection compromised investigated indices of the gut (43), and nano-selenium as the only additive did not improve the VH parameter. However, these results show that both compounds, combined under physiological or pathophysiological conditions, can act synergistically to improve intestinal barrier function. The lack of effect on VH in the duodenum and ileum is partly consistent with the study of Sopian et al. (44), who also observed no detectable effect of *Cannabis sativa* residues (CR) on this parameter. CD in the duodenum was significantly higher in the *C. perfringens* and infected groups fed a diet with CBD or nano-Se than in the control and infected and uninfected groups fed the two additives. This value was significantly lower in the jejunum in control, CBD + nano-Se, and CBD + nano-Se + *C. perfringens* groups compared to the *C. perfringens* and infected groups fed a diet with CBD or nano-Se additives. The crypt is a tubular gland formed by the small intestinal epithelium descending into the lamina propria at the root of the villi (45). The increase in CD in the infected group is consistent with a study by Guo et al. (46) showing that *C. perfringens* can damage the intestinal barrier. Additionally, the increase in CD indicates a larger necessity for cell proliferation to maintain normal intestinal health (1). This indicates that the increased depth of crypts in the infected groups may, on the one hand, be an indicator of active epithelial regeneration in response to damage. Still, on the other hand, it may also suggest chronic tissue damage and remodeling. The control group, which was neither infected nor received supplementation, showed a lower CD, which is expected in healthy intestines. Furthermore, this also indicates that the use of CBD or nano-Se alone in the diet did not show a deteriorated effect on the duodenum. The VC in the duodenum and jejunum was significantly higher in the uninfected group with CBD + nano-Se supplementation compared to the infected control group and the infected groups with CBD or nano-Se supplementation, and significantly lower in the ileum in the control group compared to the infected group with CBD and CBD + nano-Se supplementation. Higher VC indicates a higher rate of digestion and absorption function (47) but also suggests a greater need for cell proliferation to maintain intestinal integrity (48). This result is consistent with the studies of Sopian et al. (44) and Chen et al. (49), which showed that both nano-selenium and CR increased VC. In the current authors’ opinion, the presence of higher VC values indicates that the combination of CBD and nano-Se promotes improved intestinal integrity even in the absence of infection, mainly due to an increase in villi length with a similar rate of crypt depth in these groups. The lower values in the control group in the ileum indicate undisturbed mucosal indices. CBD and nano-Se in broiler diets affected the increase in VC in the ileum, which may indicate that there is a greater need for regeneration as a result of infection-induced damage in this segment and that these supplements improve the ability to regenerate the epithelium and maintain intestinal homeostasis in the presence of infection. Corresponding to the above, a reduction in VH and an increase in CD was found in the nano-Se + *C. perfringens* group in the present study. Shorter villi and deeper crypt may lead to decreased resistance to disease, lower growth performance, poor nutrient absorption, and increased secretion in the gastrointestinal tract (40, 50, 51). On the other hand, it should be noted that, as in the case of CD in jejunum, VC also did not differ between the control group and the infected group with two additives in duodenum and jejunum. This is important, as it reinforces the idea that combining CBD with nano-Se may show a protective beneficial effect on the gut barrier, protecting the chickens from the harmful effects of *C. perfringens* infection. An assessment of villi width showed that VW was lower in the CBD + nano-Se + *C. perfringens* group compared to the CBD + nano-Se group in both the duodenum and ileum. This is consistent with Zhao et al. (52), who showed a reduction in VW in *C. perfringens*-infected chickens. This shows that adding CBD and nano-Se can improve the functional status of the intestines under non-challenge conditions. On the other hand, Chen et al. (1) showed that birds with intestinal barrier failure had wider villi. According to this study, a widening of the villi indicates a smaller surface area for nutrient absorption and probably also a greater amount of gut-associated immune tissue proliferation and accumulation in the villi, which may be an indicator of impaired intestinal health. On the other hand, it is important to note that there was no significant difference between the control and the infected groups with the addition of CBD and nano-Se, supporting the above conclusion that the two components act synergistically and positively with each other under challenging conditions. Another indicator that showed significant differences was WT, which was significantly lower in the ileum in the *C. perfringens* group compared to the control group and the CBD-infected group. Reduced WT may directly result from the bacteria and their toxins, which can damage intestinal epithelial cells and lead to villous atrophy (53). The thickness of the intestinal wall under healthy conditions is the result of a balance between epithelial cell proliferation, migration, and exfoliation, which ensures the structural stability of the intestines. In contrast, the significantly greater wall thickness in the infected and supplemented group with CBD, which has anti-inflammatory and antioxidant effects, indicates a potential protective effect of this compound and may promote regeneration of damaged tissues and limit degradation of intestinal structures, leading to increased WT. In summary, gut region-specific effects have critical implications for the nutritional and immune status of broiler chickens. Changes in duodenal parameters in chickens infected and supplemented with CBD and nano-Se indicate the absence of adverse remodeling or chronic tissue damage, highlighting the protective effects of these supplements in the proximal intestine. Additionally, supplementation with the two ingredients in uninfected chickens indicates increased digestion and absorption capacity, potentially enhancing the role of the duodenum in nutrient uptake. In contrast, results in the jejunum, which is a critical site for nutrient absorption, suggest that this section benefits most from combined supplementation, particularly in maintaining intestinal integrity and absorption efficiency, in both infected and uninfected chickens. These improvements can lead to better energy and nutrient utilization, supporting growth and immune function. In contrast, the effect of the combination of CBD and nano-Se on the ileum, which is often involved in immune responses, suggests anti-inflammatory and regenerative benefits and appears to enhance immune defenses and structure in this section, which is essential for maintaining intestinal homeostasis and immunity in the presence of pathogens. This regional variability highlights the potential of CBD and nano-Se as synergistic agents to optimize nutritional and immune outcomes in broilers under physiological and pathophysiological conditions.

Light and electron microscopy analyses were performed to assess the histological structure and ultrastructure of the intestine. These studies showed that CBD + nano-Se supplementation is safe for the gut and does not cause negative qualitative changes in the histological structure of the small intestine and the ultrastructure of the jejunum. This result is partly consistent with a study by Gangadoo et al. (54), who showed in a histopathological analysis that nano-selenium did not cause harmful effects, revealing intact epithelial cells in the gastrointestinal tract in broiler chickens. This result also confirms the current authors’ previous study (32), which found that the combination of these two nutritional additives showed no opposite effect on mediating the host response to infection. This confirms that understanding the mechanisms of action of cannabidiol and nano-selenium is of great interest in developing new possibilities for promoting intestinal barrier function. In the present experiment, *C. perfringens* infection did not cause necrotic enteritis. The changes were moderate in character and mainly involved damage to the intestinal epithelium. This correlates with the clinical condition of the chickens, as none of the challenge tests induced overt clinical signs of NE. According to Timbermont et al. (16), NE is characterized by a strong inflammatory response in its early stages, where the most significant early changes are seen at the interface between the enterocyte domain and the lamina propria. An ultrastructural study by Olkowski et al. (55) shows that primary modifications occur at the level of basal and lateral domains of the enterocytes. In contrast, the apical domain of enterocytes remains intact even in the face of advanced necrotic changes. This indicates that mucosal necrosis does not result from direct damage to the mucosal epithelium. Rather, the necrotic death of enterocytes is a consequential effect of destroying lamina propria, the extra-cellular matrix, and intercellular junctions. This result is consistent with the study of Olkowski et al. (56), who also found no overt mucosal necrosis typical of NE field cases in a histological examination in *C. perfringens*-infected chickens. This is very important because the current study aimed to induce a moderate infection, mimicking a subclinical infection rather than the acute form. It should be mentioned here that increased collagenolytic enzyme activity in the mucosal and intestinal tissue environment is an integral component of the pathological process leading to NE (32, 55). However, none of the supplements administered had a remarkable effect on the morphological changes in the gut developed during infection. Perhaps it was too short a time to see changes at the microscopic level because, at the molecular level, treatment with nano-Se and CBD significantly increased the mRNA expression of genes such as junctional adhesion molecule 2 and zonula occludens-1, which are responsible for intestinal integrity (32, 33). In addition, in a previous study, the authors also showed that cannabidiol and nano-selenium in *C. perfringens*-infected chickens promoted changes in the extracellular activity of bacterial enzymes in the gut, which may indicate increased energy consumption under induced stress conditions (32).

In the current experiment, epigenetic, immunological and oxidative changes as a response to treatment in the blood and jejunum of chickens were investigated. TNF-α is an important pro-inflammatory cytokine that plays a key role in immune responses during infection. It helps initiate and regulate the immune system response by promoting inflammation, cell survival, and elimination of pathogens. However, dysregulation or overproduction of TNF-α may contribute to chronic inflammation (57, 58). In blood, significantly higher TNF-α in the CBD + *C. perfringens* group was found compared to the nano-Se + *C. perfringens* and CBD + nano-Se + *C. perfringens* groups. This may show a specific effect of this compound on the immune system. The increase in TNF-α levels may be related to an increase in the inflammatory response induced by the infection and suggests that the CBD supplement, rather than having an immunosuppressive effect, may have increased the inflammatory response in the context of this infection. Still, it should be noted that no significant difference was found between the control group and the infected group with one or two supplements. CBD is well known for its immunomodulatory effects (59, 60), but there are studies indicating that in the bacterial response, it may also induce an increase in the production of pro-inflammatory cytokines (61, 62). The increased TNF-α levels in the CBD-infected chicken group may be the organism's attempt to counteract the stress stimuli through an enhanced inflammatory response. In this study, TNF-α expression was not altered by the *C. perfringens* challenge (C vs. *C. perfringens* group), which agrees with previous studies (63–65). The difference in TNF-α levels between the group of chickens supplemented with CBD alone and the group supplemented with both nano-selenium and CBD may be due to the synergistic effects of the two compounds. Nano-selenium, through its antioxidant and immune response modulating effects, may have reduced the pro-inflammatory effects of CBD alone, explaining the lower TNF-α levels in the group with the combination of these supplements, which is consistent with the results of Sendani et al. (66). On the other hand, the lack of a significant difference between the control group and the infected group with the addition of both nano-selenium and CBD may suggest that the combination of these two compounds has a balanced effect on the immune system, mitigating the excessive inflammatory response caused by the infection, allowing for a more controlled immune response. On the other hand, in the gut, significant differences were shown in the expression of Casp 3, IgY, and % of methylated DNA. Caspase 3 is a key enzyme in the process of programmed cell death (apoptosis). Casp 3 activation is often triggered in response to various stressors, leading to controlled cell death that helps maintain homeostasis and eliminate damaged cells (67). In this study, Casp 3 levels were significantly lower in the control group compared to the *C. perfringens*, nano-Se + *C. perfringens*, and CBD + nano-Se groups. This result contrasts the study of Guo et al. (46), who found a lack of significance in Casp 3 levels in *C. perfringens*-infected chickens. In contrast, this result aligns with the study by Huang et al. (68), which showed that infections can significantly increase caspase 3. However, the increase in this factor in the CBD + nano-Se and nano-Se + *C. perfringens* groups is not easy to explain. The possible action was that nano-selenium at the dose used in the experiment exhibits a pro-apoptotic effect, perhaps to accelerate the elimination of damaged cells. It is important to note that no significant differences were shown between the control group and the CBD + *C. perfringens* and CBD + nano-Se + *C. perfringens* groups, which is consistent with a study by de Fillips et al. (69), which showed that CBD reduces inflammation in patients with LPS-induced colitis and lowers Casp 3 levels. It can also be concluded that the beneficial effect of nano-selenium, in this case, may depend on synergism with cannabidiol, or cannabidiol could have reduced the potential pro-apoptotic effect of nano-selenium. Evaluation of the immunoglobulin Y concentration showed that it was significantly lower in all test groups compared to the control and non-infected group with cannabidiol and nano-selenium. In poultry, IgY is a type of special immunoglobulin that is produced by stimulation of specific antigens *in vivo* and has a similar function to immunoglobulin G (IgG) in mammals (70). However, very limited information is available on this immunoglobulin and *C. perfringens* infection in poultry and its concentration dependence on nano-selenium and cannabidiol. Decreased IgY concentrations in chickens with bacterial infections may indicate an impaired immune response, favoring further infection and intestinal barrier damage. This is consistent with a study by Cui et al. (70), who showed reduced levels of IgY mRNA expression in the jejunum after LPS treatment of *E. coli* in chickens. Contemporary research confirms that IgY supplementation in the context of pathogen control may have a supportive effect. Still, it may sometimes be ineffective, which may explain the lower concentration of this immunoglobulin in the study groups (71, 72). Interestingly, IgY concentration in the control group and the non-infected group with the addition of CBD + nano-Se was at the same level. This indicates that the addition of cannabidiol and nano-Se does not, by themselves, reduce the concentration of IgY in chickens. The % of methylated DNA was significantly higher in the infected group compared to all other study groups, which is consistent with the results of Ognik et al. (11), which showed that *C. perfringens* infection increased the percentage of methylated DNA in the wall of the ileum in turkeys. DNA methylation is a key process regulating gene expression, which affects many cellular functions, including immune response, cell growth, and stress response. Evidence from *in vivo* and *in silico* studies shows that cannabidiol can regulate the activity of enzymes responsible for DNA methylation due to its direct binding to the enzymes and/or regulating their activity through neurotransmitter-mediated signaling (19, 73). In a rodent model of iron-induced neurodegeneration, CBD normalized mitochondrial DNA methylation levels in the hippocampus (73). Toubhans et al. (74) showed that selenium can modulate histone methylation, highlighting its important role in redox biology. Both assumptions are consistent with the current results, as the results in the infected groups with additives and the control group were at similar levels. Given the above, it may be concluded that the lack of significance in the assays of the other parameters indicative of epigenetic and oxidative changes may be related to two main facts: (i) the *C. perfringens* infection was not sufficient to induce the full changes in the parameters studied, and (ii) the exposure to challenge factor was too short to bring about the aforementioned changes fully.

In the current study, in order to evaluate the relationship between intestinal barrier functions and epigenetic and oxidative changes, a correlation analysis was performed of levels of diamine oxidase and lactic acid in the intestine and levels of the above-mentioned indicators in blood and jejunum. DAO is an intracellular enzyme in intestinal cells that catalyzes the breakdown of histamine in GIT. Its concentration under physiological conditions is relatively high in the intestine and very low in plasma (75). In contrast, lactic acid is the end product of glucose oxidation in aerobic glycolysis and can be synthesized *in situ* on the intestinal mucosa (76). Increased lactic acid production in the small intestine is a negative phenomenon, as it can lead to acidosis, which results in severe damage to the intestinal epithelium, resulting in excessive intestinal permeability, referred to as “leaky gut” (76). There were no differences between DAO concentrations and blood indices, which is consistent with the ELISA analysis results, as apart from TNF-α, no significant differences were found in the other parameters. In addition, DAO is a marker of intestinal barrier integrity, while epigenetic and oxidative indices may reflect the body's systemic response to various stressors (77). Interestingly, a statistically significant positive correlation was found between LA levels and superoxide dismutase levels. This is a beneficial effect because SOD, as an antioxidant enzyme, plays a key role in protecting cells from damage from free radicals (ROS), and there are reports that LA can affect a number of metabolic and immune processes in the body, including by affecting the redox status through the reaction of lactate dehydrogenase inducing reactive oxygen species and acting as an inhibitor of glucose breakdown (78). In the gut, the current study showed a significant positive correlation between DAO and IgY, and between LA and 8-OHdG. Schade et al. (79) highlight the roles of IgY in neutralizing toxins and pathogens, which helps maintain intestinal health. Increased DAO levels may indicate an intensification of enzymatic activity to protect against inflammation caused by pathogens or damage to the intestinal barrier, leading to activation of the local immune response, manifested by an increase in IgY. On the other hand, the association between LA and 8-OHdG may indicate that increased lactic acid production in the intestines of chickens leads to increased oxidative stress and cellular damage, as manifested by higher levels of 8-OHdG. Valavanidis et al. (80) report that 8-OHdG is one of the predominant forms of free radical-induced oxidative damage and is, therefore, widely used as a biomarker of oxidative stress and carcinogenesis. A negative correlation was also detected between DAO and IgA in the gut. Immunoglobulin A plays an important role in protection against gastrointestinal infections, supports intestinal homeostasis, and maintains the health of the intestinal microbiota (81). This may lead to the conclusion that high DAO activity may be an indicator of a healthy intestinal barrier, where protective mechanisms work effectively and reduce the need for a strong immune response in the form of sIgA. On the other hand, decreased DAO levels may suggest a weakened intestinal barrier, leading to increased production of sIgA to protect against potential pathogens.

Moreover, the results of the correlation analysis of histomorphometry vs. DAO and LA showed a significant negative correlation between villi width in the duodenum and DAO in the blood. DAO is released into the blood from mucosal cells due to damage to the intestinal barrier, leading to increased gastrointestinal permeability (46). This correlation may suggest that a reduction in the width of the intestinal villi may reflect the functional state of the barrier and is associated with damage to the intestinal wall, leading to an increase in intestinal permeability and escape of DAO into the bloodstream. A negative correlation was found between the concentration of LA in the blood and the crypt depth of the duodenum and villi width of the jejunum and ileum. Studies indicate that an increase in the synthesis of lactic acid in the intestines and the accompanying ejection of this compound into the blood can be observed in intestinal hypoxia, causing an increase in the anaerobic breakdown of glucose (82). On the other hand, correlation analysis between intestinal factor concentration and morphometry shows a positive correlation between DAO and villi height to crypt depth ratio and between LA and wall thickness and villi area of the duodenum. A positive correlation was also found in the ileum between villi width and crypt depth and DAO, as well as between villi width and LA. The correlation with DAO may be related to improved intestinal morphology and reduced intestinal barrier dysfunction and inflammation. The positive correlation between LA and parameters in the duodenum and ileum suggests that the presence of lactic acid in moderate amounts may support the healthy development and maintenance of intestinal villi, increasing intestinal absorptive capacity, and according to a study by Okada et al. (82), lactate may stimulate the proliferation of enterocytes and intestinal epithelial cells. The current study also indicated a negative correlation between DAO in the ileum and the villi height to crypt ratio in the ileum. This result is surprising and contrasts with the positive effect of DAO observed on parameters such as villi width and crypt depth. A high DAO concentration in the ileum may signal that inflammatory processes and intestinal damage are advanced enough to negatively affect the regenerative capacity and functionality of this intestinal segment, leading to a lower VH/CD ratio.

The current study evaluated the concentration of hemoglobin (Hb) in the blood of chickens. Hemoglobin is a key protein responsible for oxygen transport in chickens, and its function is fundamental to metabolic processes and homeostasis. In the context of poultry health research, measuring the concentration of hemoglobin in the blood can be an important indicator of the health status of animals, their ability to transport oxygen efficiently, as well as the body's response to various interventions, including infections or dietary supplementation. Ognik et al. (83) demonstrated that turkey hens with poorer health status were characterized by reduced hemoglobin levels, which may partly explain the significant differences between the infected group without and with nano-selenium or both supplements. The current results showed that hemoglobin concentration was significantly higher in the infected and supplemented groups with nano-selenium or the two nutritional supplements and in the uninfected group with CBD and nano-selenium, compared to the positive and negative control groups, which may suggest the beneficial effects of these nutritional supplements in increasing the blood's oxygen transport capacity and supporting erythrocyte production. Interestingly, all groups with nano-selenium supplementation had higher hemoglobin concentrations. This result is consistent with a study by Arain et al. (84), who showed increased Hb concentration in selenium-supplemented goats. However, Boostani et al. (85), who studied the effects of organic, inorganic, and nano-selenium on blood in broiler chickens exposed to oxidative stress, showed no significant differences in hemoglobin concentration between the study groups. The increased levels of the erythrocytic system markers may also be explained by the condensation of blood due to body dehydration resulting from diarrhea. However, in the current study, a subclinical infection was induced, which was not associated with clinical symptoms, such as diarrhea, which may also partly explain the lack of differences between the negative and positive control groups. On the other hand, there were no significant differences between the control group and the infected group with the addition of CBD alone. Data in the literature are quite contradictory about the effect of cannabidiol on parameters such as hemoglobin concentration. This result is consistent with several previous studies that also showed no significant differences in hemoglobin concentration and CBD supplementation in rats and goats (86, 87). However, previous studies in guinea pigs and rats demonstrated that hemoglobin concentration showed a consistent but non-significant decrease (88) or was significantly reduced (89) in the CBD groups compared to the control group. Therefore, further study is needed to determine the effects of CBD and nano-selenium on erythrocytes and hemoglobin production in chickens.

Finally, with reference to the results above, it is also important to present the performance response to treatment that we examined in our previous work (32). Body weight gain (BWG) in the experiments on days 9–23 and 24–35 was not significantly different between groups, while BWG calculated for the entire experimental period was significantly lower in the *C. perfringens*-infected group and in the CBD-infected group compared to the control group. Feed conversion ratio (FCR) was worsened in all experimental groups compared to the control group during the feeding period of days 9–23 but was not affected during other feeding periods. In contrast, feed intake was similar in all treatment groups during all periods of the study. This suggests that the response of chickens to infection is more complex than a simple increase or decrease in feed consumption and that mechanisms controlling feed utilization are implicated.

The main limitation of the present study is legislation. Currently, cannabidiol is not authorized for use as an animal feed additive in EU Member States. According to EU regulations, *Cannabis sativa* plants can be legally registered for cultivation when their tetrahydrocannabinol content is less than 0.2%. According to Commission Regulation (EU) 2022/1104 of July 1, 2022, amending Regulation (EU) No. 68/2013 on the catalog of feed materials, and with the Open Community Catalog of Feed Materials, it is permitted to use in animal feed such ingredients as hemp seed, hemp seed cake, hemp seed oil, hemp flour and hemp fiber from *Cannabis sativa L*. with a tetrahydrocannabinol content of less than 0.2% based on the method of quantification established by Regulation (EU) No. 639/2014. The European Food Safety Authority (EFSA) must approve all feed supplements. Cannabidiol is also regulated under novel food legislation. Therefore, due to a great discussion regarding application of *Cannabis* plant, future research, such as that showing the high potential of CBD, is needed to bring cannabidiol into legal use.

In conclusion, the current study provides evidence that cannabidiol and nano-selenium mediate epigenetic and oxidative DNA changes and affect intestinal development and functionality in broiler chickens at an early stage of infection with *C. perfringens.* Histomorphometry results indicate that cannabidiol and nano-selenium, in combination under physiological or pathophysiological conditions, can manifest a synergetic effect on robust intestinal barrier functions. Moreover, it was also found that the use of CBD or nano-Se alone or combined in the diet did not show an adverse effect or negative qualitative changes on the gut structures, as demonstrated by a study of ultrastructures and a histopathological examination. Moreover, it was also shown that *C. perfringens* infection did not cause necrotic enteritis, and the changes were moderate and primarily involved damage to the intestinal epithelium. This indicates that mucosal necrosis is not due to direct damage to the mucosal epithelium. Rather, necrotic death of enterocytes is a consequence of the destruction of the lamina propria, extracellular matrix, and intercellular junctions. On the other hand, a study of markers of epigenetic and oxidative DNA changes, such as TNF-α, Casp 3, IgY, or % of methylated DNA, found that the combination of these two compounds has a balanced effect on the immune system, mitigating the excessive inflammatory response caused by the infection, allowing for a more controlled immune response. This also indicates that the addition of cannabidiol and nano-Se does not reduce the concentration of IgY in chickens. In addition, the current study showed a significant correlation between DAO or LA and SOD, IgY, IgA, 8-OHdG, or histomorphometric parameters. Evaluation of hemoglobin levels demonstrates that all groups with nano-selenium supplementation had higher concentrations, and that the CBD-infected group did not differ from the control group. This study also found new mechanisms of action for cannabidiol and nano-selenium.

## Data Availability

The original contributions presented in the study are included in the article/supplementary material. Further inquiries can be directed to the corresponding authors.
